# Frailty in liver transplantation: Exploring prescribing exercise as medicine to improve patient outcomes

**DOI:** 10.1111/liv.15986

**Published:** 2024-06-20

**Authors:** Sara J. Harris, Jonathan G. Stine

**Affiliations:** 1 College of Medicine, The Pennsylvania State University, Hershey, Pennsylvania, USA; 2 Division of Gastroenterology and Hepatology, Department of Medicine, Penn State Health - Milton S. Hershey Medical Center, Hershey, Pennsylvania, USA; 3 Fatty Liver Program, Penn State Health - Milton S. Hershey Medical Center, Hershey, Pennsylvania, USA; 4 Liver Center, Penn State Health - Milton S. Hershey Medical Center, Hershey, Pennsylvania, USA; 5 Department of Public Health Sciences, The Pennsylvania State University - College of Medicine, Hershey, Pennsylvania, USA; 6 Cancer Institute, Penn State Health - Milton S. Hershey Medical Center, Hershey, Pennsylvania, USA

**Keywords:** advanced chronic liver disease, cirrhosis, nutrition, physical activity, sarcopenia

## Abstract

Liver transplantation (LT) represents a curative avenue for individuals with advanced chronic liver disease. Given the inherent illness severity of LT candidates, identifying patients at greater risk for adverse outcomes before and after transplantation is paramount. Approximately 50% of cirrhotic patients are frail and have considerable functional impairment. Various measures have been used to assess frailty, including performance-based tests and functional status evaluations. Frailty carries significant prognostic implications and predicts both mortality and pre- and post-LT complications. Contributing factors to frailty in this population include sarcopenia, malnutrition, inflammation, and psychosocial factors. Recognizing the prevalence of frailty among LT candidates, exercise interventions have been developed to improve physical frailty and offer potential to improve patient outcomes. While many interventions have demonstrated efficacy without notable adverse events, the absence of a universally accepted standard for exercise prescription underscores the variability in intervention elements and patient adherence. Given the safety profile of exercise interventions, there remains a critical need for standardized protocols and guidelines to optimize exercise regimens for LT candidates. This review delves into the landscape of frailty among LT candidates, elucidating its etiological underpinnings, impact on outcomes, utilization of exercise interventions, and the efficacy of exercise programs in reducing the burden frailty in those awaiting LT.

## 1 ∣ INTRODUCTION

Chronic liver disease (CLD) affects over 1.5 billion individuals worldwide,^1^ making it a notable health concern. While liver transplantation (LT) can provide a curative therapeutic option, an inequity remains between organ supply and patients with advanced chronic liver disease (avdCLD) awaiting LT. Additionally, patients assessed for LT tend to be older, sicker, and more medically complex given the rise of liver-related metabolic comorbidities.^2^ Thus, it is important to identify patients who are most likely to have complications and poor-outcomes before and after LT to develop interventions to minimize these complications and adverse outcomes. Currently, there are numerous models, such as the Model for End-Stage Liver Disease (MELD) 3.0, that can be used to risk-stratify and predict mortality in patients awaiting transplantation.^3^ While MELD 3.0 is more accurate compared to previous iterations, it is unable to accurately account for physical frailty, which has been reported to be even more predictive than MELD score of waiting-list drop out.^4^

Frailty was originally defined in the field of geriatrics and refers to a state of increased risk of vulnerability and poor outcomes in response to a stressor due to cumulative decline in physiologic reserves.^5,6^ When applied to hepatology and liver transplantation, frailty has been explored in the context of physical frailty which refers more to functional impairment.^2^ Frailty is associated with an increased risk in death and unplanned hospitalizations in outpatients with cirrhosis and hospitalizations in patients on the LT list,^7-9^ making it an important risk factor to assess for among LT candidates.

It is estimated that upwards of 40% of LT candidates are considered to be frail.^7,10^ Furthermore, approximately 50% of patients with cirrhosis of any aetiology have frailty, independent of their need for LT.^11^ While frailty prevalence differs based on the type of assessment, disease severity, and comorbidities,^12^ it nonetheless remains of high interest given its association with poor outcomes both before and after LT. Importantly, mortality before LT persists after adjusting for liver disease severity,^13^ and worsening frailty over time is associated with death and delisting regardless of baseline frailty.^14^ Additionally, LT recipients who are frail prior to transplantation have increased healthcare utilization including longer hospital stays, prolonged ICU stays, non-home discharges, higher number of days hospitalized in the 90 days post-transplant, and overall mortality.^15,16^ Given the ramifications of frailty for LT candidates, much research effort has focused on ways to improve frailty in this patient population.

Unlike most mortality predictors such as age, sex, and MELD 3.0 score, frailty is a modifiable risk factor among LT candidates. One method to reverse frailty is through exercise training.^17-19^ Prehabilitation programs using fitness trackers, smartphone applications and home-based exercise-programs are a safe and effective method to combat frailty.^19-22^ In recognition of this, exercise and prehabilitation programs are now recommended by the American Society of Transplantation to reduce frailty.^2^ The purpose of this review is to summarize our current understanding of the impact of frailty on LT candidate assessment and outcomes and the use of exercise training to combat frailty within this patient population.

## 2 ∣ FRAILTY ASSESSMENT IN LIVER TRANSPLANT CANDIDATES

Multiple tools have been used to assess frailty in LT candidates. These tools include the 6-minute walk test (6MWT), Activities of Daily Living (ADL), Braden scale, cardiopulmonary exercise testing (CPET), Clinical Frailty Scale (CFS), Fried Frailty Phenotype (FFP), gait speed, Instrumental Activities of Daily Living (IADL), Karnofsky Performance Status (KPS), Liver Frailty Index (LFI), Short Physical Performance Battery (SPPB), and gait speed.^2^ Functional exercise capacity is assessed by CPET and 6MWT.^23^ CPET, the gold-standard for determining cardiopulmonary reserve, measures maximal oxygen consumption at peak exercise (VO2 peak) and thus aerobic capacity.^24^ A lower cardiopulmonary reserve is associated with pre- and-post LT mortality.^25,26^ Lower VO2 peak in LT candidates was associated with increased mortality within 100 days of LT, lower one-year survival, longer hospital stays post-transplantation, and a longer supplemental oxygen requirement post-transplantation.^27,28^ The 6MWT can also evaluate functional status with shorter distances associated with increased risk of mortality, hospitalizations, and health-related quality of life.^29-31^ Additionally, the 6MWT was found to be a better prognostic indicator than sarcopenia as it assesses muscle function rather than size.^29^ While CPET and the 6MWT provide objective data to assess frailty, other tools such as the FFP and LFI can be used more readily as they do not require specialized settings and only need minimal expertise to complete.

Mainly studied in the outpatient setting, the FFP and LFI have been used to assess frailty in LT candidates quite frequently. The FFP defines frailty as decreased physiologic reserve as measured by having three of the following: (1) low physical activity; (2) unintentional weight loss of ten pounds in one year; (3) slow walking speed; (4) grip strength weakness; and (5) self-reported exhaustion.^5^ Higher FFP scores are associated with increased waitlist mortality,^13^ unplanned hospitalizations,^7^ and longer hospital stays.^8^ The LFI is a quick performance-based measure of physical function that consists of hand grip strength, chair stands, and balance and is one of the most comprehensive and liver-specific tools used.^32^ The LFI improves mortality prediction compared to clinician assessment alone or MELDNa alone.^4,33^ The LFI is reproducible,^34^ can be performed at bedside, and can be tracked longitudinally allowing it to be a useful assessment.^35^ However, the LFI does not include an exercise capacity component, which is a strong predictor of waitlist morality.^26^ The Duke Activity Status Index (DASI) is a validated questionnaire that can predict exercise capacity.^36^ It was recently tested as a predictor of LT candidate outcomes and demonstrated it predicts pre-transplant and overall mortality in patients with avdCLD.^37,38^ Both the DASI and LFI are low cost assessments that can be performed quickly.^38^

Similar to the performance-based LFI, the SPPB, ADL/IADL, and KPS scales are provider-and-patient-based metrics of frailty. These tools are advantageous as they can be completed systematically in both outpatient and inpatient settings. The performance-based SPPB includes balance testing, chair stands, and gait speed and predicts LT waitlist mortality.^13,39^ Next, ADL and IADL are self-reported scales that assess disability and are also validated to predict mortality.^40^ When used in the context of frailty, disability represents how frailty impacts daily activities. Both ADL and IADL can predict waitlist mortality independent of MELD score.^40^ A different assessment, KPS, is a provider administered measurement of functional status with poor performance status associated with increased mortality, especially after hospital discharge.^41-43^ Another subjective tool is the CFS which assesses patients in nine categories on a scale ranging from terminally ill to very fit.^7^ It is an effective tool to predict mortality regardless of muscle mass,^44^ unplanned hospitalizations, and death.^7^ Next, gait speed as a measurement of frailty is an independent risk factor for cirrhosis complications leading to hospitalizations.^9^ Lastly, the Braden Scale, which is used for pressure ulcer risk and is administered to all hospitalized patients in the United States, predicted postoperative LT complications such as non-home discharges and greater hospitalization length of stay.^16^

Assessing frailty in LT candidates is not without challenges and limitations. CFS, KPS, and Braden scales require clinician judgement while CPS, KPS, ADL/IADL, Braden scale, and FFP can be influenced based on patient reports (Table 1). Additionally, only roughly one-third of the tools are responsive to changes over time including LFI, SPPB, gait speed, and grip strength.^2^ While KPS is more cost effective, its subjective nature has led to poor inter-rater reliability. Similarly, the subjective nature of ADL/IADL warrants caution with their use; however, they are quick tools that are cost-effective and can be done at bedside.^45^ Despite being the gold standard measurement for exercise capacity, CPET use is limited by cost, required staff training, and possible patient discomfort in the setting of ascites.^37^ SPPB requires trained individuals to assess patient walking speed, thus limiting its use to patients who are able to walk. Lastly, required time to perform a particular assessment and ease of completing an assessment may limit the ability to assess frailty in LT candidates. Therefore, quick, more cost-effective methods may be more well-suited and we should continue to strive to create a tool that satisfies these important criteria.

## 3 ∣ IMPACT OF FRAILTY ON LIVER TRANSPLANTATION OUTCOMES

Frailty impacts LT outcomes in the short-term and long-term. First, physical decline leading to frailty may result in removal of a patient from the LT list and premature death. Frailty is also associated with increased hospitalizations, longer hospital stays, a longer supplemental oxygen requirement post-LT, non-home discharges, and poor health-related quality of life.^8,28,31^

Frailty also affects long-term LT outcomes including patient and graft survival following LT. Prior to LT, it is well-known that individuals with advCLD who have higher 6MWT distances and VO2 peak have significantly higher rates of survival compared to those with shorter 6MWT distance and lower VO2 peak.^46^ Additionally, worsening frailty is associated with delisting and death regardless of baseline MELD score,^14^ highlighting the importance of combating frailty assessment prior listing and transplanting patients with avdCLD. Furthermore, KPS scores prior to and after LT independently predict patient and graft survival.^47^ This is congruent with a systematic review which found that severe frailty led to a reduction in early survival by two fold and late survival by 50%,^48^ a finding which may be explained by the fact that frailty is associated with acute cellular rejection within three months post-LT despite similar immunosuppressive treatments.^49^ In another cohort of LT recipients, frailty increased the odds of acute cellular rejection by over three fold.^50^

**FIGURE 1:**
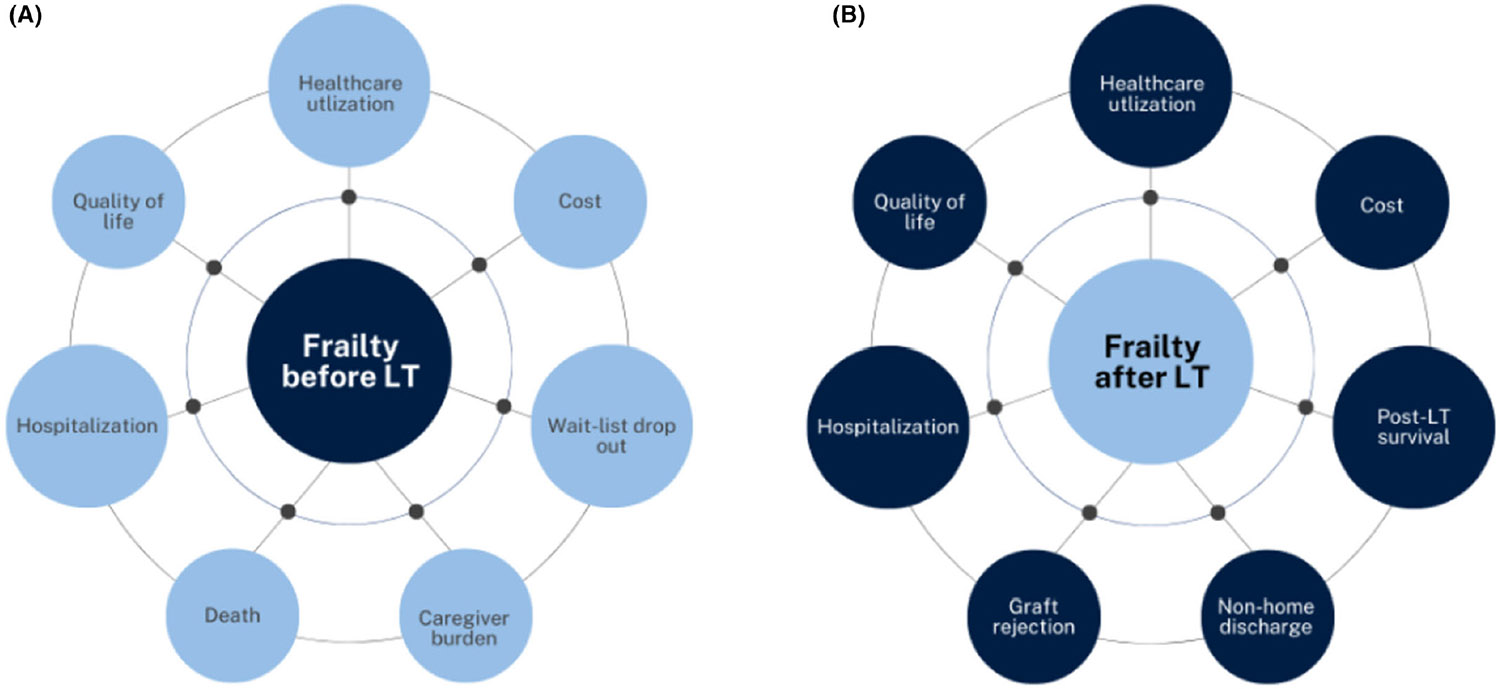
Implications of frailty before (A) and after liver transplantation (B).

Taken together, the increased short- and long-term adverse outcomes create a strain on the healthcare system with greater rates of both healthcare utilization and costs.^51^ Transplant costs are disproportionately increased in patients with worse performance status. In the three months prior to LT, costs are tripled for patients with worse functional status. Furthermore, in the first year post-transplant, patients with worse functional status accrued >40% more costs.^52^ Patients with frailty use more resources leading to more of an economic burden on the healthcare system. Figure 1 summaries the impact of frailty on patient outcomes both before and after liver transplantation.

## 4 ∣ MECHANISMS UNDERLYING FRAILTY IN LIVER TRANSPLANT CANDIDATES

By understanding the mechanisms that lead to the development of frailty it may be possible to develop targeted interventions to combat frailty and these subsequent worse LT outcomes and added costs.

Numerous mechanisms underlie the development of frailty in liver transplant candidates. First, liver disease related factors such as sarcopenia, malnutrition, and inflammation contribute to frailty. Sarcopenia is defined as a loss of muscle mass and strength^53^; however, in the context of cirrhosis is often referred to solely as the loss of muscle mass.^12^ Similarly to frailty, sarcopenia is a poor prognostic factor.^54,55^ Malnutrition refers to an imbalance (either deficiency or excess) in nutrient consumption.^56^ Sarcopenia, malnutrition, and inflammation all play a complex role in the development of frailty.

Sarcopenia can lead to frailty through several mechanisms. Liver dysfunction results in hyperammonemia which upregulates myostatin expression thus inhibiting skeletal muscle mass, increasing reactive oxygen species production, and causing mitochondrial dysfunction. As a result, this hinders protein synthesis while augmenting proteolysis.^57,58^ Also, patients with cirrhosis have lower levels of circulating testosterone which impairs protein synthesis as testosterone usually stimulates muscle development and maintenance.^59,60^ In fact, lower testosterone levels are a worse prognostic indicator for men with cirrhosis and have been associated with increased mortality and need for LT.^61^ Hormonal balance further impacts sarcopenia as patients with cirrhosis may have insulin resistance which can also lead to muscle mass loss.^62^ Additionally, cirrhosis decreases ghrelin and increases leptin levels which together can alter energy balance causing muscle wasting and contributing to malnutrition.^63,64^ Other causes of malnutrition leading to frailty include ascites causing early satiety, maldigestion, disruption in gut bacteria, and encephalopathy.^65^ Gut microbial dysbiosis can occur via inflammation mediated methods and will be discussed below.

The next major mechanism of frailty is secondary to CLD causing chronic inflammation. Proinflammatory mediators, such as cytokines, are released in response to hepatic necrosis and endotoxemia.^66^ Due to impaired gut barrier function, altered gut bacterial diversity, portosystemic shunts, and impaired function of Kupffer cells, bacterial endotoxins are able to circulate in the blood triggering a proinflammatory cascade.^67-70^ These inflammatory mediators lead to elements of frailty such as anorexia, fatigue, muscle wasting, and energy deficiency.^12^ Also, chronic inflammation induces hepatic dysfunction resulting in impaired muscle synthesis, and it induces anorexia leading to malnutrition. Lastly, hepatic encephalopathy can augment the appearance of frailty due to fatigue, immobility, altered taste perception, and decreased energy expenditure.

## 5 ∣ PSYCHOSOCIAL FACTORS THAT PLAY A ROLE IN FRAILTY DEVELOPMENT IN LIVER TRANSPLANT CANDIDATES

Psychosocial factors are associated with frailty. In patients with cirrhosis, frailty was associated with increased pain, depression, and worse overall health-related quality of life,^71^ and is often independent of liver disease severity.^10^ Additionally, health-related quality of life is inversely related to mortality in patients with advCLD, again independent of MELD score,^72^ highlighting the importance of screening patients for psychosocial factors of frailty independent of disease progression. Resilience is another psychosocial factor that is inversely related with frailty. Low resilience is associated with increased frailty in patients with cirrhosis.^73^ While psychosocial factors are not included in the physical frailty definition they are important components to consider when assessing overall patient frailty and interventions that improve these factors are recommended to routinely be incorporated in prehabilitation programs at LT centres.^74^

## 6 ∣ EXERCISE PRESCRIPTION FOR LIVER TRANSPLANT CANDIDATES AND ITS IMPACT ON PATIENT OUTCOMES

Physical activity level is a modifiable risk factor for frailty in LT candidates^75^ and thus offers promise to improve outcomes in LT candidate.^76^ LT candidates are often deconditioned due to complications from their advCLD which can lead to limited aerobic capacity, frailty, sarcopenia, and malnutrition in this patient population.^77,78^ While regular physical activity in LT candidates can prevent frailty and maintain cardiorespiratory fitness independent of MELD score,^79^ most individuals are sedentary. In fact, only one-third of the daily steps and average amount of time in moderate to vigorous activity are completed by those with advCLD and 76% of waking hours are spent in sedentary behaviour.^80^ There is a clear unmet need for those with advCLD to reduce sedentary behaviour and increase their physical activity.

Importantly, multiple studies have demonstrated exercise training can reduce frailty.^19,21^ Other studies have shown significant benefit in cardiorespiratory fitness, muscular fitness and health-related quality of life as summarized in Table 2. To date, various exercise types have been studied, including aerobic exercise, resistance training and balance exercises to name a few. Aerobic exercises may include cycle ergometry, walking or even a pedal boat.^19,20,81,82^ Free weights and elastic bands have been used for resistance training.^19,83^ Importantly, many studies have used a combination of aerobic and resistance training. Fitness trackers, smartphone applications and home-based exercise-programs have all been tested through the STRIVE, EL-FIT, MATRIX and PRIMER studies, with varying degrees of efficacy and adherence.^19-22^ At least eight weeks duration is what appears necessary to lead to clinically meaningful improvement in patient outcomes based on expert opinion. Table 3 summarizes the FITT principles of exercise prescription that should be considered in clinical trial design and also prehabilitation programs that include an exercise component.

Regardless of the exercise type or the delivery platform, all studies have demonstrated exercise training to be safe in individuals with advCLD, including those awaiting liver transplantation.^84^ Historically there has been concern that acute exercise could increase portal hypertension and subsequently induce gastroesophageal variceal haemorrhage.^85^ However, over time as one becomes physiologically acclimated to the stress of regular exercise training, the opposite is seen and exercise training programs have been shown to reduce portal pressures^86,87^ and prevent hepatic decompensation.^83^ However, despite this promising safety data, it should be highlighted that to date, the large body of research in the field of exercise training and advCLD excludes individuals with the most advanced disease and those with MELD >20 or Child-Turcotte-Pugh Class C disease are largely excluded from published clinical trials (Tables 2 and 3).

Besides ensuring safety, another challenge to exercise programs is participant adherence. Across exercise programs, attrition and adherence rates can vary. Attrition rates have ranged from 5% to 36% with adherence rates spanning between 14% and 100% and higher in supervised activity sessions (Table 2). A systematic review of eight studies found adherence to exercise programs of 38%–90% in unsupervised programs and higher than 94% in supervised.^88^ Barriers to adherence may include liver related symptoms, fatigue, and weather.^22^ While adherence rates are higher in supervised programs, the feasibility of these programs is limited by patient time commitment, costs, and transportation.^89^ Therefore, the use of wearable trackers that allow for self and provider monitoring in LT candidates can provide more accurate measures of adherence and may be easier to implement due to the ability for remote monitoring.

While remote monitoring of exercise may help with patient adherence, there are other factors that impact one's ability to exercise. First, fatigue is a common theme that has emerged across the scientific literature. Patients with Child-Pugh Class C reported exercise was significantly more fatiguing and difficult than patients in Classes A/B.^90^ Additionally, LT candidates commonly self-report fatigue to be a significant barrier to exercise.^91^ Other health conditions have been recognized as potential limitations to exercise such as hepatic encephalopathy and ascites amongst others.^91^ Interestingly when asked, caregivers of patients with cirrhosis, report pain and underlying medical conditions to be the most significant challenges that precluded patients under their care from exercising. Social factors also have been self-reported to limit the ability of individuals with CLD to perform physical activity, including work, household chores, and childcare duties.^92^ Moreover, patients who underwent solid organ transplants have cited additional barriers to include physical limitations, motivation, a lack of guidance, no access to facilities, risk aversion and cost preventing regular physical activity.^93,94^

## 7 ∣ CHALLENGES AND FUTURE DIRECTIONS

While exercise programs have been shown to be effective, barriers exist to implementing exercise interventions in clinical practice. First, there is a lack of consensus on which single frailty assessment tool should be used. Each tool currently available has its own advantages and disadvantages and as we look to improve upon what is currently available, designing a novel tool that is (1) valid and reliable; (2) sensitive and liver-specific; (3) feasible and accessible; (4) multidimensional and comprehensive; (5) cost-effective; (6) scalable to the entire population with advCLD; and (7) readily implemented into routine clinical practice will remain paramount. To date, the LFI is the tool that comes closest to checking each of these necessary boxes. Second, our current approach to exercise prescription is a one-size-fits-all approach and lacks individualization. In an era where personalized medicine is becoming increasingly common, exercise prescription in which each element of the FITT principals can be varied to achieve a specific cumulative weekly dose of exercise would seem advisable, especially if it can be tailored to overcome individual barriers to regular physical activity which are identified on an individual level prior to undertaking an exercise training program. Third, what is the role of technology? Will exercise programs that rely on digital therapeutics with smartphone applications or fitness trackers lead to improved adherence and thus greater efficacy? Or will this be an environment that is already wrought with inequity and access issues that become more problematic and favour those with greater socioeconomic means where this equates to better digital healthcare literacy and access to technology? Lastly, what is the role of exercise prescription in individuals with the most advanced CLD? To date, those with MELD >20 and CPT Class C disease are excluded from clinical trials and both the safety and efficacy of exercise training remains unknown in these populations. Unfortunately, many times this is the population most in need of intervention and future trial design should consider how to best tailor an exercise program to address this key unmet need. If addressed, this may prevent the considerable waiting-list drop-out in this population where one-fourth of individuals awaiting LT will be removed from the wait-list at some point in time.

Once a novel frailty tool is developed, it is crucial to consider how it should be implemented into practice. Should frailty be a standardized tool included in LT evaluation? We argue that it should be as frailty drastically impacts outcomes both pre- and post-transplant. Across studies frailty has been associated with increased risk of death and unplanned hospitalizations and worsening frailty is associated with waiting-list removal. Frailty also leads to increased healthcare utilization and costs related to longer hospital and ICU stays in the immediate perioperative period, non-home discharges, and an increased number of days hospitalized in the later post-LT period. Furthermore, in the long term, worse functional status is associated with higher overall costs post-LT owing in part to increased healthcare utilization. Lastly, frailty is known to reduce graft survival, increased the odds of T-cell mediated rejection, and decreased patient survival. Therefore, in our opinion, every patient undergoing evaluation for LT should be assessed for frailty, and frailty should be used as a criterion for transplant selection and considered as an additional factor alongside the current LT selection process. While severe frailty can become a contraindication for transplantation, there remains no agreed upon threshold with the currently available tools that objectively measure physical frailty where LT would be considered futile. We look to future research to answer this question. Lastly, at this point in time, it appears LT may not fully reverse frailty in light of the fact that physical frailty continues to persist well after the short-term perioperative period following LT. Additional studies are needed to better understand why physical frailty persists long-term after LT and to design effective interventions to combat this highly significant problem.

## 8 ∣ CONCLUSION

Frailty remains common in individuals awaiting lifesaving LT and negatively impacts patient outcomes before and after liver transplantation. Exercise training programs offer promise to reverse frailty and thus maintain or improve an individuals' chances of LT and should be offered to all individuals as a standard element of prehabilitation programs available at most LT centres worldwide.

## Figures and Tables

**TABLE 1:** Tools currently available to screen for frailty in LT candidates.

Tool	Measures	Objective/Subjective
CPET	Functional capacity: Maximal O2 consumption, Aerobic capacity, Cardiopulmonary reserve	Objective—performance based
6MWT	Functional capacity: Distance walked in six minutes	Objective—performance based
LFI	Hand grip strength Chair stands Balance	Objective—performance based
Gait speed	Velocity Time to walk predetermined distance	Objective—performance based
SPPB	Chair stands Balance testing Gait speed	Objective—performance based
FFP	Physiologic Reserve: Physical activity Unintentional weight loss in one year Walking speed Grip strength weakness Self-reported exhaustion	Objective—performance based Subjective—self reported, can be biased based on patient report
DASI	Exercise capacity: 12-item physical activity	Subjective—can be biased based on patient report
ADL/IADL	Disability: Ability to feed, dress, bathe, toilet	Subjective—can be biased based on patient report
KPS	Functional status	Subjective—can be biased based on patient report, requires clinician judgement
CFS	Fitness: Nine category scale ranging from terminally ill to very fit	Subjective—can be biased based on patient report, requires clinician judgement
Braden scale	Pressure ulcer risk	Subjective—can be biased based on patient report, requires clinician judgement

Abbreviations: 6MWT, six minute walk test; ADL, activities of daily living; CFS, Clinical Frailty Scale; CPET, cardiopulmonary exercise testing; DASI, Duke Activity Status Index; FFP, fried frailty phenotype; IADL, independent activities of daily living; KPS, Karnofsky Performance Score; LFI, liver frailty index; SPPB, short performance physical battery.

**TABLE 2:** Exercise-based clinical trials in individuals with advCLD.

Study (year)	Sample size	advCLD severity	Exercise intervention	Adherence	Patient outcomes improved
Limongi et al.^95^	17 (5 Ex, 12 Con)	CTP A/B/C: NR MELD: 18 ± 5	Freq: 7d/wk. Int: Moderate Type: RT Time: 3 sets of 15 reps 12 wks., home-based, unsupervised	NR	CRF HRQOL Muscular fitness
Zenith et al.^82^	19 (9 Ex, 10 Con)	CTP A/B/C (%): 74/26/0 MELD: 10 ± 2	Freq: 3d/wk. Int: Moderate-to-vigorous Type: AT Time: 40 min 8 wks., facility-based, supervised	NR	CRF HRQOL Muscular fitness
Roman et al.^96^	20 (10 Ex, 10 Con)	CTP A/B/C (%): 81/13/6 MELD: 9 ± 2	Freq: 3d/wk. Int: Moderate Type: AT Time: 60 min 12 wks., facility-based, supervised	Adherence: 83% Drop out: 20%	CRF HRQOL Muscular fitness
Limongi et al.^97^	45 (22 Ex, 23 Con)	CTP A/B/C: NR MELD: 18 ± 6	Freq: 7d/wk. Int: Moderate Type: RT Time: 3 sets of 15 reps 12 wks., home-based, unsupervised	Adherence: NR Drop out: 36%	CRF HRQOL Muscular fitness
Macias-Rodriguez et al.^98^	29 (14 Ex, 15 Con)	CTP A/B/C (%): 64/36/0 MELD: 11 ± 3	Freq: 3d/wk. Int: Moderate-to-vigorous Type: AT + RT Time: 30–40 min 14 wks., facility-based, supervised	Adherence: 97% Drop out: 21%	CRF HRQOL Muscular fitness
Roman et al.^83^	25 (15 Ex, 10 Con)	CTP A/B/C (%): 100/0/0 MELD: 8 ± 1	Freq: 3d/wk. Int: Moderate Type: AT Time: 60 min 12 wks., facility-based, supervised	Adherence: 94% Drop out: 7%	CRF Muscular fitness
Kruger et al.^99^	40 (20 Ex, 20 Con)	CTP A/B/C (%): 70/30/0 MELD: 9 ± 3	Freq: 3d/wk. Int: Moderate-to-vigorous Type: AT Time: 30–60 min 8 wks., home-based, unsupervised	Adherence: 55% Drop out: 5%	CRF Muscular fitness
Wallen et al.^100^	17 (8 Ex, 9 Con)	CTP A/B/C (%): 38/62% B or C MELD: 13 ± 4	Freq: 3d/wk. Int: NR Type: AT + RT Time: NR 8 wks., facility-based supervised or home-based unsupervised	Adherence: 75–95% (higher for in-person) Drop out: 10%	Muscular fitness
Aamann et al.^101^	39 (20 Ex, 19 Con)	CTP A/B/C (%): 50/50/0 MELD: 10 ± 3	Freq: 3d/wk. Int: Moderate Type: RT Time: 60 min 12 wks., facility-based, supervised	Adherence: 82% Drop out: 5%	CRF HRQOL Muscular fitness
Chen et al.^18^	17 (9 Ex, 8 Con)	CTP A/B/C (%): 0/78/22 MELD: 17 ± 4	Freq: 7d/wk Int: Moderate Type: AT (fitness trackers with step goals) Time: NR 12 wks., home-based, unsupervised	Adherence: 100% Drop out: 11%	CRF Muscular fitness
STRIVE study^21^	83 (58 Ex, 25 Con)	CTP A/B/C (%): 44/ 54 B or C MELD: 14 ± 4	Freq: 3d/wk. Int: Moderate Type: RT (fitness videos with bands) Time: 30 min 12 wks., home-based, unsupervised	Adherence: 14% Drop out: 26%	Frailty
EL-Fit study^20^	28 (28 Ex)	CTP A/B/C (%): 32/ 69 B or C MELD: 19 ± 5	Freq: NR Int: Low-to-Moderate Type: AT + RT (smartphone app) Time: NR 38 ± 12 d, home-based, unsupervised	Adherence: 77% Drop out: 11%	CRF
MATRIX study^19^	21 (21 Ex)	CTP A/B/C (%): 0/52/48 MELD: 17 ± 5	Freq: NR Int: Moderate Type: AT + RT (smartphone app) Time: NR 12 wks., home-based, unsupervised	Adherence: 57% Drop out: 24%	CRF Frailty
PRIMER study^22^	30 (20 Ex, 10 Con)	CTP A/B/C (%): NR MELD: 13	Freq: 3d/wk. Int: Moderate Type: AT (fitness trackers, step goals) Time: NR 14 wks., home-based, unsupervised	Adherence: 50% Drop out: 10%	CRF

Abbreviations: App, application; AT, aerobic training; Con, control; CPT, Child-Turcotte-Pugh; CRF, cardiorespiratory fitness; Ex, exercise; Freq, frequency; HRQOL, health-related quality of life; Int, intensity; NR, not reported; Reps, repetitions; RT, resistance training.

**TABLE 3:** FITT principles of exercise prescription for individuals with advCLD.^a^

	Aerobic exercise training	Resistance training
Frequency (weekly)	3–5 days	2 days
Intensity	Moderate (50%–70% HRR^b^) or Vigorous (>70% HRR)	Moderate Muscular strength^c^: 50%–85% 1-RM Muscular endurance^c^: <50% 1-RM
Time	150 min/wk. for Moderate intensity 75 min/wk. for Vigorous intensity	Muscular strength: 2–4 sets of 8–12 repetitions for each of the major muscle groups for muscular strength Muscular endurance: 12–20 repetitions instead
Type	Prolonged, rhythmic activities targeting large muscle groups (e.g., walking, cycling, swimming)	Resistance machines, free weights, and/or body weight exercises^d^

Abbreviations: HRR, heart rate reserve; 1-RM, one repetition maximum.

a FITT principles have largely been studied in advCLD patients who have MELD ≤ 20 and CTP Class A or B.

b If a heart rate monitor is not readily available, the talk test is suggested to estimate the intensity of exercise, where an individual should be able to talk but not sing comfortably.^102^

c Muscular strength is the force generated with a single effort while muscular endurance refers to the capacity to perform voluntary muscular contraction for a prolonged period.

d Low impact exercises such as body weight squats or alternating lunges may be prescribed for injury prevention.

## Key points

- Frailty is common in LT candidates and is associated with increased risk of mortality and adverse events, such as hospitalizations, increased hospital stays, and decreased post-LT survival.
- Many measurement tools exist to assess for frailty, including the liver-specific Liver Frailty Index.
- Liver disease complications such as sarcopenia, malnutrition, inflammation, and immunosuppression contribute to frailty. Psychosocial factors also play a strong role.
- Physical inactivity is a modifiable factor that can reduce the risk of frailty through exercise training.
- Although there is no consensus on the ideal exercise prescription, interventions using aerobic and/or resistance training are safe and effective in improving frailty.

## Abbreviations:

ADL: Activities of Daily Living
advCLD: advanced chronic liver disease
CSF: Clinical Frailty Scale
CPET: cardiopulmonary exercise testing
DASI: Duke Activity Status Index
FFP: Fried Frailty Phenotype
IADL: Instrumental Activities of Daily Living
KPS: Karnofsky Performance Status
LFI: Liver Frailty Index
LT: liver transplantation
MELD: Model for End-Stage Liver Disease
SPPB: Short Physical Performance Battery
6MWT: 6-minute walk test
